# Targeting TRP Channels for Pain, Itch and Neurogenic Inflammation

**DOI:** 10.3390/ijms25010320

**Published:** 2023-12-25

**Authors:** Ari-Pekka Koivisto, Arpad Szallasi

**Affiliations:** 1 Pain Therapy Area, R&D, Orion Pharma, 20360 Turku, Finland; 2 Department of Pathology and Experimental Cancer Research, Semmelweis University, 1083 Budapest, Hungary

Transient receptor potential (TRP) channels are multifunctional signaling molecules with important roles in health and disease. More than two decades of intensive preclinical and clinical research supports the involvement of TRP channels in pain, itch and neurogenic inflammation. Through this Special Issue, we aimed to gain insights into the broad range of on-going research activities focused on both established and emerging targets for TRP channel modulators. To our pleasant surprise, the COVID-19 pandemic did not slow down the steady progress in this expanding field as evidenced by the 11 papers that this Special Issue attracted. 

The majority of our knowledge on TRP channel structure and function is based on information gathered over the past 25 years. Following the discovery of the Drosophila TRP channel, the search for homologues in the early 1990s revealed the existence of mammalian TRP channels. At this time, the role of TRP channels in sensory perception was not appreciated. This changed dramatically with the molecular cloning and functional expression of the capsaicin (vanilloid) receptor TRPV1 by David Julius and colleagues in 1997 [1]. Subsequently, several other TRP channels involved in the itch and pain pathways were identified. In 2021, David Julius was awarded the Nobel prize in Medicine and Physiology for his groundbreaking work on the molecular identification of receptors for temperature and touch: specifically, for the identification of TRPV1 as a noxious heat-sensing ion channel, and TRPM8 as the cold-sensing menthol receptor [1,2]. The 2021 Nobel prize was shared with Ardem Patapoutian, predominantly for his work on mechanically activated Piezo channels as touch receptors [3]. However, Patapoutian and his team have also made fundamental contributions to TRPA1 and TRPM8 research by cloning TRPM8 [4] and TRPA1 [5] and by establishing covalent binding as a unique activation mechanism of the TRPA1 protein [6].

Soon after a number of TRP channels had been identified in pain- and itch-sensing neurons, the question of the redundancy of TRP channel expression in pain and itch sensing emerged, an important question that still keeps scientists baffled. Pain and itch scientists were asking the same question: would blocking a single TRP channel provide clinically meaningful analgesia and itch/scratch relief, or should we aim to block several TRP channels at the same time? Whereas small molecule TRPV1 antagonists turned out to be disappointing analgesic drugs in clinical trials, on-going studies with the ultrapotent TRPV1 agonist resiniferatoxin in osteoarthritis pain (Grunenthal, Aachen, Germany) and vocacapsaicin (Concentric Analgesics, San Francisco, CA, USA) in postoperative pain support the concept that multi-ion channel blocking via sensory neuron desensitization can achieve clinically meaningful pain relief. However, both resiniferatoxin and capsaicin evoke an initial pain reaction which limits their clinical value. 

The next-generation sequencing (NGS) of human genomes provides a combination of genetic and phenotypic data that is boosting drug discovery efforts by providing human-relevant efficacy and safety signals. The initial report that TRPM8 is linked to migraine [7] is now confirmed by UK and FinnGEN Biobank data [8,9,10], strongly supporting the role of TRPM8 in migraine pathogenesis. The first clinical study in healthy volunteers with a selective TRPM8 antagonist by Pfizer revealed that TRPM8 antagonists are able to alleviate cold pressor test-induced pain. However, a few volunteers suffered from unwanted side-effects that may be either compound- or mechanism-related [11]. Further, the selective TRPM8 antagonist did not reduce core body temperature in humans [12]. Preclinical validation of TRPM8 as a migraine target shows that genetic knockout of TRPM8 protects from nitroglycerin-induced headache, but it was not possible to alleviate chronic migraine pain via pharmacological inhibition of TRPM8 [13]. Whether or not animal models of migraine headache predict clinical efficacy remains to be seen. It also remains to be seen if TRPM8 should be directly blocked by an antagonist or functionally desensitized with a partial agonist. 

A better understanding of TRPM8-expressing neuronal circuits and other tissues involved in migraine pain signaling with the help of carefully validated antibodies by Hernandez-Ortego et al. is a step closer toward that goal [14]. Spekker et al. have written a timely and balanced review of the pre-clinical and clinical data on the roles of TRPM8, TRPV1, TRPV4 and TRPA1 in migraine [15]. Cohen et al. [16] wrote an excellent review on current anti-migraine drugs and discussed TRPC4 as an emerging new target for the treatment of migraine pain.

A new look into the redundancy of TRP channels as pain, inflammation and itch transducers is afforded by single-cell RNA sequencing data. The emerging classification of sensory neuron subtypes revealed that the peptidergic and non-peptidergic dichotomy established in mice is probably not valid in human [17]. Instead, several pain-sensing sensory neuron subtypes and a specific population of pruritogen receptor-enriched sensory neurons detecting itchy stimuli are found in human dorsal root ganglia [18]. Interestingly, about 60% of human sensory neurons express TRPV1, a much larger proportion than in rodents. Sadler et al. showed that about 75% of human sensory neurons express TRPC5 ion channels and that TRPC5 is most likely activated in multiple pain models and conditions [19]. Bernal et al. showed that TRPC5 in odontoblasts signal cold pain in teeth [20]. Taken together, these results suggest that, despite being co-expressed in some sensory neuron populations, TRPV1 and TRPC5 channels have non-redundant roles and that their selective block is sufficient to provide analgesia. 

The expression of TRP channels in cell types mediating innate (such as macrophages, dendritic cells, keratinocytes, mast cells and natural killer cells) and adaptive (lymphocytes) immune responses suggests involvement in the regulation of inflammation. Further, neuronally expressed TRP channels are shown to regulate neurogenic inflammation. Ludwig et al. show for the first time that dihydroceramide, a membrane component of Gram-negative bacteria, sensitizes the TRPV1 ion channel. This novel finding may partly explain why bacterial inflammation causes pain in wound infections [21]. Keratinocytes and mast cells are shown to sensitize pruritogen receptor-expressing sensory neurons and cause itch and scratching behavior [22]. Um et al. review the participation of the TRPV3 ion channel in itch [23]. TRPV3 is expressed in keratinocytes and gain-of-function mutations cause chronic itch in mice and men. There is a compelling reason to believe that selective block of TRPV3 could alleviate chronic itch. Mahdmoud et al. cover the pre-clinical and clinical literature on TRP channels and itch. They also discuss an important topic that is often neglected or forgotten, namely the intriguing ethnic differences in TRP channel responses [24].

Osteoarthritis is a common and disabling chronic joint disease. Osteoarthritis prevalence is increasing globally, driven by obesity [25]. Existing pharmacotherapies for treatment of osteoarthritis are relatively poor, and osteoarthritis is the primary reason for joint replacement surgery, accounting for more than 90% of operations. Anti-NGF antibody showed robust analgesic efficacy in several osteoarthritis pain clinical trials but its development as a drug was terminated due to rapidly progressing osteoarthritis in a subset of patients [26,27]. A more thorough understanding of NGF signal transduction and detailed mechanism of action of how anti-NGF antibody therapy causes cartilage and bone injury are hot topics that can potentially lead to the identification of novel and safe therapies. Interestingly, Halonen et al. provide evidence that chondrocytes from osteoarthritis patients express nineteen different TRP channel genes [28]. The authors provide intriguing evidence that TRPA1 and TRPM8 agonists induce matrix metalloprotease expression, a mechanism that could play a role in chondrocyte injury. Here, a disease-modifying potential of TRPA1 and TRPM8 antagonists for treatment of osteoarthritis is emerging.

In summary, the papers comprising this Special Issue provide an excellent overview of the preclinical promise of drugs targeting TRP channels for pain and itch relief, along with the challenges that these compounds may encounter in clinical practice. The potential therapeutic opportunities for drugs that target TRP channels are expanding. But some caution is indicated here. Experimental evidence for TRP channel function in certain sensory pathways is based on the actions of compounds of questionable selectivity or staining by antibodies of uncertain specificity. It is our hope that Special Issues like this will help distinguish between on-target and off-target TRP drug actions. 
